# Supplementary material for: AgRP1 modulates breeding season-dependent feeding behavior in female medaka
Source: eLife. 2025 May 29;13:RP100996. doi: 10.7554/eLife.100996 (PMC12122003; doi:10.7554/eLife.100996)
Supplement: Figure 1—source code 1. [file elife-100996-fig1-code1.zip › eLife-VOR-RA-2024-100996/Figure1Source code1.pdf]

```
#Copyright Kimura Tomoki & Shinji Kanda @ Dept.Biol.Sci, Grad.Sch.Sci, The University of
Tokyo
#This script can be run under python 3.5. OpenCV3 should be installed.
#a key add the total number
#z key reset the total number
#q key quit the program
#space key pauses the program
#this script can be run with 3 parameters like python thisfilename.py 5 3 1

import sys
import numpy as np
import cv2
import time
def conv(frame, argvs):
    gray = cv2.cvtColor(frame, cv2.COLOR_BGR2GRAY)
    #binarization.INV means black and white inversion. blockSize means pixel cutoff.  3, 5, 7,etc.
    th1 = cv2.adaptiveThreshold(gray,255,cv2.ADAPTIVE_THRESH_GAUSSIAN_C,¥
            cv2.THRESH_BINARY_INV,29,8)
    #iterations – number of times dilation is applied.anchor – anchor of the kernel that indicates
the relative position of a filtered point within the kernel; the anchor should lie within the kernel;
default value (-1,-1) means that the anchor is at the kernel center.
    kernel = np.ones((int(argvs[1]),int(argvs[1])),np.uint8)
    dilation = cv2.dilate(th1, kernel, iterations =1)
    erosion = cv2.erode(dilation,kernel,iterations = int(argvs[2]))
    dilation = cv2.dilate(erosion, kernel, iterations =int(argvs[3]))
    return dilation
#draw a circle after detemining the center of each shape
def CoG(contours,frame):
    arr=[]
    for i in contours:
        x_=0
        y_=0
        for j in i:
            x_ += j[0][0]
            y_ += j[0][1]
        arr.append([int(x_/len(i)), int(y_/len(i))])
    arr = np.array(arr)
    for i in arr:
         cv2.circle(frame,tuple(i), 5, (0,255,0), 1)
def count(frame,gray):
    #edge detection
    img, contours, hierarchy =
cv2.findContours(gray,cv2.RETR_EXTERNAL,cv2.CHAIN_APPROX_SIMPLE)
```

```
        CoG(contours,frame)
        #img = cv2.drawContours(frame, contours, -1, (0,255,0), 1)

        return contours
totalNum = 0
lastLog = 0
pressCount = 0
argvs = sys.argv
argc = len(argvs)
if (argc != 4):
        print ('Usage: python count.py [kernel] [erosion] [dilation]¥nkernel...Increase the parameter to reduce the noize. It increases the possiblity to misditection of the object')
        print('erosion...Increase this value when you want to distinguish the objects conted as one.¥ndilation...Increase this value when one object is double counted. Too large value may cause multiple objects counted as one')
        quit()              # Program End
cap = cv2.VideoCapture(0) #0 is the number of the camera

print('fps={0:2d}, w={1}, h={2}'.format(int(cap.get(cv2.CAP_PROP_FPS)),cap.get(3), cap.get(4)))
print('kernel={0}, iterations of erosion={1}, iterations of dilatioin={2}'.format(int(argvs[1]),int(argvs[2]),int(argvs[3])))
while(cap.isOpened()):
        ret, frame = cap.read()

        if ret:
                fin_img=conv(frame, argvs)
                contours=count(frame,fin_img)     #Edge detection,   draw circles
                cv2.putText(frame,"realtime#="+str(len(contours)),(10,40), cv2.FONT_HERSHEY_COMPLEX, 1.0,(0, 0, 255))       #print number of object
                descrip = "space:pause q:quit"
                cv2.putText(frame,"space:pause q:quit a:add z:reset",(10,440), cv2.FONT_HERSHEY_COMPLEX, 0.5,(0, 255, 0))       #print number of object
                cv2.putText(frame,"total#=" + str(totalNum) + "(" + str(pressCount) + ")",(10,80), cv2.FONT_HERSHEY_COMPLEX, 1.0,(0, 0, 255))       #total number
                cv2.imshow('frame',frame)     #Out put 1 frame in the window
        else :
                break
        keycode = cv2.waitKey(1)       #wait 1ms for key input
        if keycode == ord('q'): #when q is pressed, program ends
                break
        elif keycode == ord(' '):     #when space key was pressed
                while(True):
                        keycode = cv2.waitKey(0)       #wait key press forever
```

```
            if(keycode== ord(' ')):     #space key: pause
                break
            elif(keycode== ord('q')): #q key stops the program
                cap.release()
                out.release()
                cv2.destroyAllWindows()
                quit()
    elif keycode== ord('a'): #when a key is pressed,add the number to the total number
        if time.time() > lastLog+1: # avoid multiple count in a short time
            totalNum = len(contours) + totalNum
            lastLog = time.time()
            pressCount = pressCount+1

    elif keycode== ord('z'): #when z key is pressed, reset the total number and press count.
            totalNum = 0
            pressCount = 0


cap.release()
cv2.destroyAllWindows()
```

Figure 1-Source code 1: The code of “Shrimp-counter” system
